# Genome-Wide Identification and Comparative Analysis of Conserved and Novel MicroRNAs in Grafted Watermelon by High-Throughput Sequencing

**DOI:** 10.1371/journal.pone.0057359

**Published:** 2013-02-26

**Authors:** Na Liu, Jinghua Yang, Shaogui Guo, Yong Xu, Mingfang Zhang

**Affiliations:** 1 Laboratory of Genetics Resources & Functional Improvement for Horticultural Plant, Department of Horticulture, Zhejiang University, Hangzhou, People’s Republic of China; 2 Key Laboratory of Horticultural Plant Growth, Development & Quality Improvement, Ministry of Agriculture, Hangzhou, People’s Republic of China; 3 National Engineering Research Center for Vegetables, Beijing Academy of Agriculture and Forestry Sciences, Key Laboratory of Biology and Genetic Improvement of Horticultural Crops (North China), Beijing, People’s Republic of China; The University of Queensland, Australia

## Abstract

MicroRNAs (miRNAs) are a class of endogenous small non-coding RNAs involved in the post-transcriptional gene regulation and play a critical role in plant growth, development and stresses response. However less is known about miRNAs involvement in grafting behaviors, especially with the watermelon (*Citrullus lanatus* L.) crop, which is one of the most important agricultural crops worldwide. Grafting method is commonly used in watermelon production in attempts to improve its adaptation to abiotic and biotic stresses, in particular to the soil-borne fusarium wilt disease. In this study, Solexa sequencing has been used to discover small RNA populations and compare miRNAs on genome-wide scale in watermelon grafting system. A total of 11,458,476, 11,614,094 and 9,339,089 raw reads representing 2,957,751, 2,880,328 and 2,964,990 unique sequences were obtained from the scions of self-grafted watermelon and watermelon grafted on-to bottle gourd and squash at two true-leaf stage, respectively. 39 known miRNAs belonging to 30 miRNA families and 80 novel miRNAs were identified in our small RNA dataset. Compared with self-grafted watermelon, 20 (5 known miRNA families and 15 novel miRNAs) and 47 (17 known miRNA families and 30 novel miRNAs) miRNAs were expressed significantly different in watermelon grafted on to bottle gourd and squash, respectively. MiRNAs expressed differentially when watermelon was grafted onto different rootstocks, suggesting that miRNAs might play an important role in diverse biological and metabolic processes in watermelon and grafting may possibly by changing miRNAs expressions to regulate plant growth and development as well as adaptation to stresses. The small RNA transcriptomes obtained in this study provided insights into molecular aspects of miRNA-mediated regulation in grafted watermelon. Obviously, this result would provide a basis for further unravelling the mechanism on how miRNAs information is exchanged between scion and rootstock in grafted watermelon, and its relevance to diverse biological processes and environmental adaptation.

## Introduction

The primary objective of horticultural industry has been to increase yield and productivity, in order to provide the vegetables needed by a growing world population during the past years. However, due to limited availability of arable land and high market demand for off-season vegetables, crops production is continuously performed on unsuitable conditions in parts of the world. These unfavourable conditions include environments that are too drought, soil salinity, extreme temperatures, and increased incidence of pests and soil-borne diseases like fusarium wilt caused by *Fusarium* spp [1]. Due to these conditions, various physiological and pathological are disordered leading to severe crop losses. One environment-friendly technique for avoiding or reducing losses in production is grafting [1], [2]. Grafting is the union of two or more pieces of living plant tissue that they grow as a single plant. In fact, grafting is a routine technique in continuous cropping systems in many parts of the word. In the past, grafting was used with vegetable crops to limit the effects of soil pathogens [3]. It was first commonly used by grafting watermelon [*Citrullus lanatus* (Thunb.) Matsum.and Nakai] onto pumpkin [*Cucurbita moschata* Duchesne ex.Poir] rootstocks in Japan during the late 1920s. Soon after, watermelons were grafted onto bottle gourd [*Lagenaria siceraria* (Molina) Standl.] rootstocks [1]. Over the past few years the demands of grafting has been increased dramatically since grafting could be used to induce tolerance to high-salinity, drought, and low temperature, which are three common environmental stress factors that seriously influence plant growth and development worldwide. In addition, graft may be used to enhance nutrient uptake, improve alkalinity tolerance, limit the negative effect of boron, copper, cadmium, and manganese toxicity and etc. [4]–[7]. Moreover, many previous studies showed that grafting also had big impacts on plant growth and development processes [8]. For example, the rootstock-scion combination may alter the amounts of hormones produced, influence on sex expression and flowering order of grafted plants. For example, compared with other rootstocks, watermelon grafted onto bottle gourd causes early formation of female flowers. In addition, grafting also has positive effects on vegetable quality such as improvement of physical properties, flavour and health-related compounds in the product [9].

The rootstock-mediate enhancement of abiotic and biotic stresses tolerance, increased yield and quality undoubtedly provide an additional motivation for vegetable grafting in modern horticulture. The physiological processes implicated in these events of grafted plants have received much attention, however, the molecular processes involved remain relatively unknown. Clearly, associated gene expression must be regulated at the transcriptional, post-transcriptional and post-translational levels in grafted plants. However, it is largely unknown how the genes or loci expression are regulated and how small RNAs are involved in the regulation.

Endogenous small RNAs (sRNAs) are 20–30 nt RNA molecules that modulate gene expression at the transcriptional and posttranscriptional levels, also roles in developmental and physiological processes in eukaryotic organisms [10], [11]. In plants, two main categories of small regulatory RNAs are distinguished based on their biogenesis and function: microRNAs (miRNAs) and small interfering RNAs (siRNAs) [12]. MiRNAs are 20–24 nt single-stranded RNA molecules that are processed from single-stranded RNA precursors that fold into stem-loop structures. This structure is then processed by Dicer-like 1 (DCL1) to produce a double-stranded RNA duplex. The duplex is exported into the cytoplasm by HASTY and methylated at the 3′ end by HUA ENHANCER 1 (HEN1) [13]. Mature miRNA is then loaded into the RNA-Induced Silencing Complex (RISC) to target mRNAs for cleavage in a sequence-specific manner. Plant miRNAs recognize their targets through near-perfect complementarity to direct RISC-mediated cleavage, although in some cases translational inhibition and epigenetic changes such as DNA and histone methylation can be the mode of action of miRNA-mediated gene silencing [14]–[16].

Currently, functional analysis of miRNAs has revealed their involvement in multiple biological and metabolic processes in plants. MiRNAs regulate various aspects of developmental processes such as auxin signaling, meristem boundary formation and organ separation, leaf development and polarity, lateral root formation, transition from juvenile-to-adult vegetative phase and from vegetative-to-flowering phase, floral organ identity, and reproduction, etc. [17]. For example, miR156 target 11 of the 17 *SPL* genes in *Arabidopsis*; among these genes, *SPL*3, 4, and 5 promote vegetative phase change as well as floral transition, and *SPL*9 and *SPL*15 regulate plastochron length [18], [19]. Several miRNAs target genes involved in auxin signaling, for example miR160, miR167 and miR393 [20]. The regulation of *ARF*s by miR160 appears to be important in many aspects of shoot and root development [21]. Besides their roles in growth, development and maintenance of genome integrity, miRNAs are also important in plant to cope with stress response such as biotic and abiotic stimuli [17], [22]. Sunkar and Zhu [23] reported that miR393, miR402, miR397b, and miR319c were induced by at least one of the treatments including drought, cold, salt and ABA, whereas miR398 was down-regulated. Further study showed that miR398 is transcriptionally down-regulated to release its suppression of Superoxide dismutase 1 (CSD1) and Superoxide dismutase 2 (CSD2) genes to improve plants tolerance to oxidative stress in *Arabidopsis* [24].

Initially, miRNAs were identified by conventional low-scale sequencing of cDNA clones from small RNA libraries prepared from different plant sources. Increasing evidence shows that a large number of known miRNAs are evolutionarily conserved across different species within the plant kingdom [12]. However, it was also reported that some miRNAs were absent in diverse species and with low-level expression, suggesting that they have evolved more recently and non-conserved [25]. Recently, the application of high-throughput sequencing technologies, such as Roche 454, ABI SOLiD and Illumina Solexa, provide a powerful strategy to identify, as well as quantify miRNAs at unprecedented perspectives [26]–[29]. With the availability of next generation sequencing technologies, it may be possible to make new discoveries of species-specific or low expressed miRNAs.

Watermelon (*Citrullus lanatus* L.) is one of the 20 most important food and agricultural commodities worldwide, and China is the highest producing country of watermelon and produces more than half the world’s watermelon (http://faostat.fao.org). Approximately 20% of watermelons in China are grafted [1]. Recently analytical technology has revealed that some specific RNA molecules can transport through phloem tissue as genetic information to execute coordinated organ development programs and the plant’s response to both environmental cues and pathogen challenge [30]. Grafting has been exploited in the field of plant physiology to clarify the mechanism of long-distance transport of specific RNAs [31]–[33]. However, roles of miRNAs in grafting watermelon have never been reported. Here we performed high-throughput sequencing and bioinformatics of grafted watermelon (watermelon was grafted on to bottle gourd and squash rootstocks, watermelon grafted on to watermelon used as control) to identify known and novel miRNAs, as well as miRNAs expression patterns when grafted on to different rootstocks. Our finding in this study may provide a basis for further investigation of the physiological function of identified miRNAs and the regulatory roles of miRNAs in rootstock-mediate effects on plant growth and development in watermelon.

## Results

### High-throughput Sequencing of Watermelon Small RNAs

**Figure 1:**
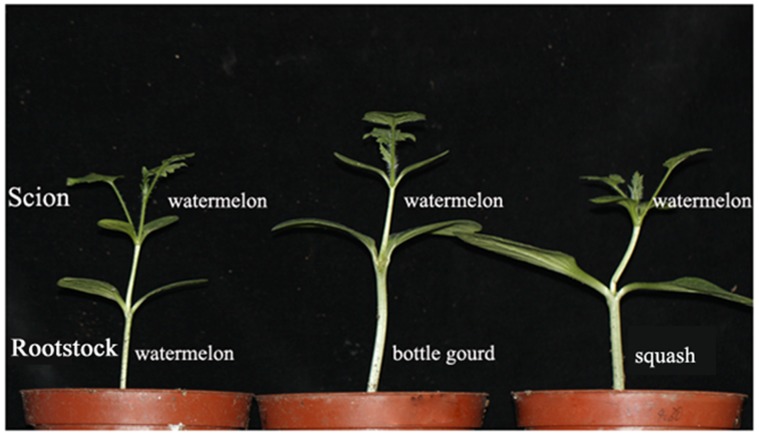
Watermelon plants grafted onto watermelon (Wm/Wm) or onto bottle gourd (Wm/BG) and squash (Wm/Sq) at the two true-leaf stage.

In order to study the role of miRNAs in grafted watermelon, watermelon (*Citrullus lanatus* L. cv. IVSM9), an inbred line was grafted onto two rootstocks: Bottle gourd ‘Yongzhen’ (*Lagenaria siceraria*) (abbreviated as Wm/BG), and squash ‘Shintozwa’ (*Cucurbita maxima*×*Cucurbita moschata*) (Wm/Sq), using ‘insertion grafting’ method as described by Lee [3], and watermelon plants grafted onto watermelon (Wm/Wm) were used as control (Fig. 1). These two rootstocks were selected because they are the most representative commercial ones used in China. Three separate small RNA libraries (Wm/Wm, Wm/BG and Wm/Sq) were generated from the part above cotyledons of the scion. Solexa data have been deposited into the NCBI/GEO database with accession number GSE33209.

**Figure 2:**
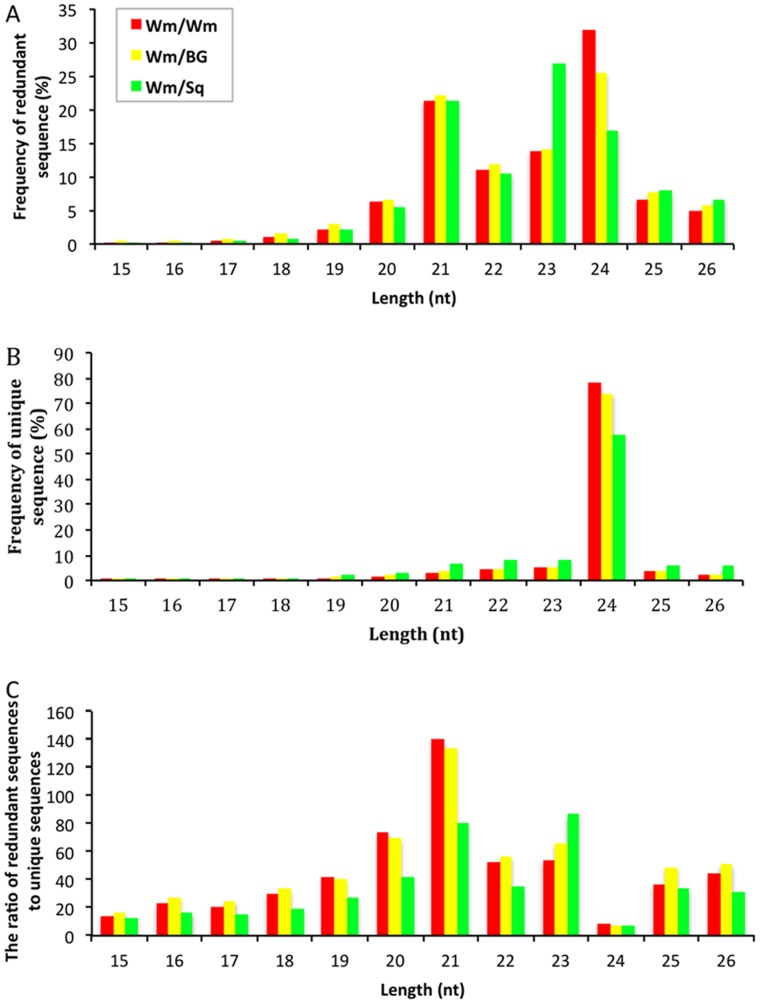
Sequence length distribution in Wm/Wm, Wm/BG and Wm/Sq.

A total number of 11,458,476 (Wm/Wm), 11,614,094 (Wm/BG) and 9,339,089 (Wm/BG) raw reads consisting of 2,957,751 (Wm/Wm), 2,880,328 (Wm/BG) and 2,964,990 (Wm/BG) unique sequences (a particular sequence with non-redundancy) were obtained through Solexa sequencing (Table 1). After further removal of rRNAs, tRNAs, snRNAs, snoRNAs, mRNA and repeat region, a total of 3,728,264, 3,583,615, 2,053,475 sRNA sequences for Wm/Wm, Wm/BG and Wm/Sq were obtained, respectively. The remaining sequences were subjected to analyse the length distribution (Fig. 2A and Fig. 2B). The majority of the redundant reads were in the range of 21 to 24 nt in length, approximately 78.29%, 73.54% and 75.90% in Wm/Wm, Wm/BG and Wm/Sq, respectively. Among which, sequences with length of 24 nt were shown to be significant in abundance in Wm/Wm and Wm/BG; however, the most abundant sequences were 23 nt long in Wm/Sq. The length distribution of unique sequences showed that sequences with length of 24 nt were shown to be significant in abundance and accounted for 78.12%, 74.13% and 57.53 of the sequences number in Wm/Wm Wm/BG and Wm/Sq, respectively. This result was consistent with that previously reported from other plant species such as *Arabidopsis* [34], *Oriza sativa* [35], *Citrus trifoliate* [36] and *Cucumis sativus* [29] in which 24 nt sRNAs dominate the sRNA transcriptome. To further compare the average abundance of sRNAs with different length, we measured the ratio of raw and unique sequences. sRNAs varied widely in length, and there is variation in their redundancies, among which the 21 nt sRNAs showed the highest redundancies in Wm/Wm (Fig. 2C). Similar phenomenon was also observed in Wm/BG, however, Wm/Sq exhibited a little difference, in which the sRNAs class with both 21 and 23 nt showed the highest redundancies. The observation indicated the differential expression pattern of distinct categories of sRNAs in the sample, and differences in the complexity of the three small RNA pools indicating different regulation underlying the miRNA-mediated effects of graft on plant growth and development.

**Table 1:** Reads abundance of various classification of small RNAs in Wm/Wm, Wm/BG and Wm/Sq.

RNA class	Wm/Wm		Wm/BG		Wm/Sq		
	Raw reads	Unique reads	Raw reads	Unique reads	Raw reads	Unique reads	
total	11,458,476	2,957,751	11,614,094	2,880,328	9,339,089	2,964,990	
mRNA	640,995	18,760	765,778	20,184	347,227	12,525	
rRNA	3,829,221	13,883	3,890,603	16,005	3,428,871	13,309	
tRNA	91,857	2,685	98,194	2,962	43,055	1,763	
snRNA	991	133	1,186	159	755	98	
snoRNA	4,425	229	4,542	270	2,392	181	
repeats	3,723,153	21,983	3,636,087	25,345	3,373,366	22,845	
Mappable	3,728,264	192,088	3,583,615	160,330	2,053,475	80,448	

### Identification of Known miRNAs

In order to investigate known miRNAs in watermelon, the unique sequences were compared with the known miRNAs in miRBase 17.0. Based on sequence homology our analysis revealed that 39 conserved miRNAs, belonging to 30 miRNA families (Table 2). There were 32, 36 and 31 conserved miRNAs were identified in Wm/Wm, Wm/BG and Wm/Sq, respectively. Among these miRNA families, 33 miRNAs were expressed in at least two of our three small RNA libraries, and 27 miRNAs were found to be shared by all three sRNA libraries. Most of the known miRNAs (79.5%) were 21 nt in length with the remaining being 20 or 23 nt long (Table 2). This result was consistent with current understanding that canonical miRNAs are 21 nt length, while canonical siRNAs are 24 nt length [37].

**Table 2:** Conserved miRNAS in watermelon.

miRNA family	Name	sequence (5′→3′)		reads(Wm/Wm)	reads (Wm/BG)	reads (Wm/Sq)	precursor	MiRNA* sequenced	Homology by specie				
									A. thaliana	M. truncatula	P. trichocarpa	V. vinifera	O. sativa
**miR156**	miR156f	TTGACAGAAGATAGAGAGCAC	21	1495	1186	524	Y	Y	√	√	√	√	√
**miR159**	miR159a	TTTGGATTGAAGGGAGCTCTA	21	97973	73719	26273	N	N	√	√	√	√	
**miR160**	miR160a	TGCCTGGCTCCCTGTATGCCA	21	2481	3181	2830	Y	Y	√	√	√	√	√
**miR162**	miR162a	TCGATAAACCTCTGCATCCAG	21	103	107	44	Y	Y	√	√	√	√	√
**miR164**	miR164a	TGGAGAAGCAGGGCACGTGCA	21	1254	1614	353	Y	Y	√	√	√	√	√
**miR166**	miR166a	TCGGACCAGGCTTCATTCCCC	21	47770	55967	39253	N	Y	√	√	√	√	√
**miR167**	miR167	AGATCATGTGGCAGTTTCACC	21	0	9	0	Y	N			√	√	
	miR167a	TGAAGCTGCCAGCATGATCTA	21	638	566	23960	Y	N	√	√	√	√	√
	miR167c	TGAAGCTGCCAGCATGATCTGG	22	89803	101182	0	Y	Y	√	√	√	√	√
**miR168**	miR168a	TCGCTTGGTGCAGGTCGGGAA	21	1326	1142	825	N	Y	√		√	√	
**miR169**	miR169a	CAGCCAAGGATGACTTGCCGG	21	209	235	251	Y	N	√	√	√	√	√
	miR169c	TGAGCCAAGGATGACTTGCCG	21	17	7	0	Y	N	√	√	√	√	
	miR169n	TAGCCAAAGATGACTTGCCTG	21	12	0	0	Y	N					
**miR171**	miR171a	TTGAGCCGTGCCAATATCACG	21	20	47	652	Y	N	√	√	√	√	√
	miR171c	TGATTGAGCCGTGCCAATATC	21	1805	1729	627	Y	N	√	√	√	√	√
**miR172**	miR172	GGAATCTTGATGATGCTGCAG	21	206	262	43	Y	N	√		√	√	√
**miR319**	miR319a	TTGGACTGAAGGGTGCTCCCT	21	8653	24804	0	N	Y					√
	miR319c	TTGGACTGAAAGGAGCTCCT	20	1200	1106	0	N	Y					
**miR390**	miR390a	AAGCTCAGGAGGGATAGCGCC	21	461	599	332	Y	Y	√	√	√	√	√
**miR393**	miR393	TCCAAAGGGATCGCATTGATC	21	220	177	60	Y	Y	√	√	√	√	√
**miR395**	miR395a	CTGAAGTGTTTGGGGGAACTC	21	430	216	6	Y	N	√	√	√	√	√
**miR396**	miR396	TTCCACAGCTTTCTTGAACTT	21	9	11	798	Y	Y	√	√	√	√	√
	miR396a	TTCCACAGCTTTCTTGAACTG	21	3906	323	8	N	N	√	√	√	√	√
**miR397**	miR397	TCATTGAGTGCAGCGTTGATG	21	72	37	4	Y	N	√		√	√	√
**miR398**	miR398a	TGTGTTCTCAGGTCACCCCTT	21	130	5444	277	Y	N	√	√	√	√	√
	miR398b	TGTGTTCTCAGGTCGCCCCTG	21	0	0	8	Y	N		√	√	√	√
**miR399**	miR399a	TGCCAAAGGAGAGTTGCCCTG	21	0	0	3	Y	N	√	√	√	√	√
	miR399g	TGCCAAAGGAGAATTGCCCTG	21	0	3	0	Y	N			√	√	√
**miR408**	miR408	ATGCACTGCCTCTTCCCTGGC	21	8865	4017	288	N	Y	√		√	√	√
**miR477**	miR477a	CTCTCCCTCAAGGGCTTCTA	20	0	11	19	Y	N					
**miR530**	miR530b	TGCATTTGCACCTGCACCTT	20	29	40	24	Y	N					
**miR827**	miR827	TTAGATGACCATCAACAAACG	21	83	59	15	Y	N	√		√		
**miR894**	miR894	CGTTTCACGTCGGGTTCACC	20	6005	6896	6250	N	N					
**miR1310**	miR1310	GGCATCGGGGGCGTAACGCCCCT	23	0	4	0	Y	N					
**miR166l**	miR166l	TCGGACCAGGCTTCATTCCTC	21	0	1972	2621	N	N	√	√	√	√	√
**miR2111**	miR2111a	TAATCTGCATCCTGAGGTCTA	21	3064	3899	2741	N	N				√	
**miR2911**	miR2911	GGCCGGGGGACGGGCTGGGA	20	6785	7658	5590	Y	Y					
**miR2916**	miR2916	TGGGGACTCGAAGACGATCATAT	23	78	88	174	Y	Y					
**miR4414**	miR4414	AGCTGCTGACTCGTTGGCTC	20	15	8	24	Y	N					

Y, yes; N, no.

In high-throughput sequencing, the relative expression level of a unique miRNA can be measured by the frequency of its read count. According to this principle, we found miRNAs had a very broad range of expression which varied from thousands even tens of thousands sequence reads to less than 10 sequence reads (Table 2). In the 30 miRNA families, miR159, miR166, miR167 and miR319 had the highest number of redundancies with more than 10,000 reads. For example, miR159a has approximately 96,576 reads in Wm/Wm, also abundant with 72,460 reads in Wm/BG and 26,273 reads in Wm/Sq. Additionally, 12 miRNA families namely miR156, miR160, miR164, miR168, miR171, miR396, miR398, miR408, miR894, miR166l, miR2111 and miR2911 were found to have some thousands reads while 6 families (miR162, miR169, miR172, miR390, miR393, miR2916) had more than one hundred reads. In comparison with the highly-expressed miRNAs, the remaining families (miR395, miR397, miR530, miR827, miR399, miR477, miR1310, miR4414) were infrequently sequenced (less than 100). It is possible that these miRNAs may be expressed at low levels in certain tissue and under specific condition. The varied frequency of sequencing among miRNA families might suggest their distinct physiological roles in watermelon development.

MiRNAs comprise both conserved and species-specific miRNA species. To identify the 30 miRNA family homologs, we did a homology search approach in a number of diverse plant species (Table 2). As expected, most of the miRNAs identified were highly conserved in diverse plant species. We speculate that the ancient regulatory pathways mediated by evolutionary conserved miRNAs may be also involved in watermelon plants. Further analysis revealed that conserved miRNAs had relative high abundant in general. For example, the most abundant one miR159 is reported highly conserved miRNA that restricts the expression of some MYB transcription factors and is involved in the regulation of vegetative growth, flowering time, anther development, seed shape and germination [38], [39]. While, some other miRNAs (such as miR477, miR530, miR1310, miR4414) were found in fewer diverse plants with relatively low expression patterns, indicating that they have specificity in a narrow range of plants. These miRNAs are classified as less conserved homologs with their function not yet clear.

### Identification of Novel miRNAs

**Figure 3:**
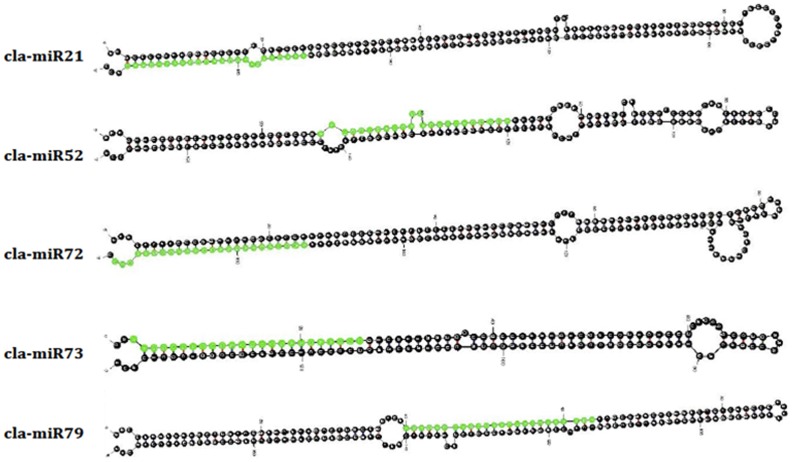
RNA secondary structure of the hairpin forming precursors of cla-miR21, cla-miR52, cla-miR72, cla-miR73 and cla-miR79. The putative mature miRNA sequences are shaded in green. Nucleotide positions are numbered starting from 5′ end of the precursor sequence.

One of the most important features of high-throughput sequencing is that it can be used to detect novel miRNAs in sRNA transcriptome [26]. Based on the available watermelon genome sequence, we identified flanking sequences of the watermelon candidate miRNAs and predicted their possibility of forming characteristic hairpin structures. According to the described criteria and methods, our analysis revealed 80 putative novel or watermelon-specific miRNAs candidates, named temporarily in the form of cla-miR number (a list with intramolecular folding capacity that resembled the characteristic of miRNA precursors see Table S1). The folded secondary structures of five candidates are shown in Fig. 3 as examples. In accordance with that reported in other plant miRNAs, our newly identified miRNAs derived from predicted hairpin structures were ranged from 69 to 259 nt. The negative folding free energies of these pre-miRNA hairpin structures ranged from −218.5 to −17.5 (kcal mol^−1^) with an average of about −59.2 kcal mol^−1^, and minimal folding free energy index (MFEI) ranged from 0.9 to 2.3 with an average of 1.2, which is apparently higher than that for tRNAs (0.64), rRNAs (0.59), and mRNAs (0.62–0.66). The described miRNAs were potentially generated from 83 different loci (Table S1) and almost all of them (except for cla-miR1 and cla-miR2) were from a single locus, consistent with most species-specific miRNA identified in other plant species.

The predicted novel miRNAs exhibited much lower expression levels, consistent with the notion that non-conserved miRNAs are often expressed at a lower level than conserved miRNAs. The majority of novel miRNAs were sequenced less than 100 reads. The low abundance of novel miRNAs might suggest a specific role for these miRNAs under various growth conditions, in specific tissues, or during developmental stages. Whether these low abundant miRNAs are expressed at higher levels in other tissues and organs, or whether they are regulated by environmental stresses, remain to be investgated.

### Confirmation of Predicted miRNAs by Stem-loop RT-qPCR

**Figure 4:**
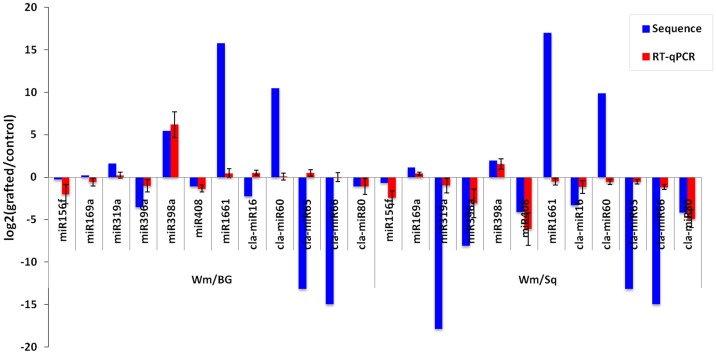
Expression analysis of miRNAs by RT-qPCR. RT-qPCR confirming the express pattern of miRNAs.

To verify the existence and expression patterns of the miRNAs from the high-throughput sequencing, several known and novel miRNAs with different expression patterns from Solexa sequencing results were selected for RT-qPCR analysis. As shown in Fig. 4, the expression patterns of the selected miRNAs obtained by RT-qPCR was similar to results from high-throughput sequencing; however, some non-conserved and novel miRNAs were identified with low read number or undetectable by Solexa sequencing, such as miR319a, miR1661, cla-miR59, cla-miR60, cla-miR65 and cla-miR66, all of them were detected by RT-qPCR (Fig. 4). It is possibly because of differences in the sensitivity and specificity between RT-qPCR and high-throughput sequencing technology. Except for the very low-abundantly expressed miRNAs, the expression patterns obtained from the RT-qPCR were consistent with the deep sequencing data.

### Prediction of miRNA Targets

MiRNAs regulate expression of specific gene via hybridization to mRNA transcripts to promote RNA degradation, inhibit translation or both. To better understand the biological functions of the known miRNAs in watermelon, putative target genes were predicted using the plant small RNA target prediction tool (TagetFinder 1.6) and all the annotated transcripts of watermelon restored in ICuGI as the reference set. Putative targets were found for the majority of the miRNAs. The predicted targets appear to be involved in a wide range of biological processes and most of them were classified as transcription factors such as SBP (miR156), MYB (miR159), ARF (miR160, miR167), NAC (miR164), MADS-box (miR164), NFY (miR169), GRAS (miR171), AP2 (miR172), TCP (miR319), GRF (miR396) and WRKY70 (miR4414) and functional proteins such as F-box protein (miR169, miR390, miR393), Ubiquitin-like proteins (miR396), ARGONAUTE 1 (miR168), Sulfate adenylyltransferase (miR395), Phosphate transporter (miR399) in plant metabolism and environmental stress response (Table S2). We found most miRNA targets are conserved across several plant species, reinforcing the idea that conserved plant miRNAs are involved in essential biological processes.

Unlike conserved miRNAs, the targets of only 11 novel miRNAs were successfully predicted (Table S3). The target genes of novel miRNAs were not enriched in transcription factors. Functional annotation of the predicted target genes showed that they were with diverse functions, ranging from genes encoding enzymes involved in metabolism, genes regulating transport, genes encoding various kinases and genes regulating oxidative reduction. The target prediction and annotation of the miRNAs can provide some new insight into how watermelon miRNA regulate gene expression.

### Expression Pattern of miRNAs among Wm/Wm, Wm/BG and Wm/Sq

**Figure 5:**
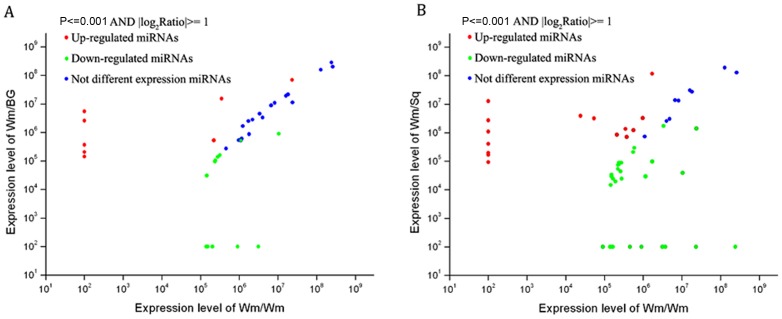
Comparison of expression patterns of miRNAs identified between Wm/Wm and Wm/BG (A) as well as Wm/Wm and Wm/Sq (B).

The biogenesis of miRNAs must be highly regulated, as suggested by the fact that many miRNAs accumulate in a spatiotemporally regulated manner or in response to environmental stimuli. Differentially expressed miRNAs between libraries give a clue to molecular events related to the plant responses to grafting with different rootstocks. We first normalized the read density measurement and then used *P*-value <0.001 and the absolute value of log_2_ratio fold-change >1.0 as a threshold to judge the statistical significance of miRNA expression. We found a total of 17 known miRNA families were significantly differentially expressed in at least one graft system compared with self-grafted watermelon (Fig. 5; Table S4). There were 5 miRNA families which were co-regulated in both graft systems, among them, miR396 and miR408 were highly reduced, while miR398 were highly induced in both Wm/BG and Wm/Sq. Notably, miR1661 was detected in Wm/BG (read count 1,972) and Wm/Sq (read count 2,621) but not in Wm/Wm, while miR319a was induced in Wm/BG and undetected in Wm/Sq. The expression patterns of all these five co-regulated miRNAs were verified by performing stem loop RT-qPCR (Fig. 4), except for miR1661 and miR319a, which were undetected by Solex sequencing in Wm/Wm and Wm/Sq respectively, were consistent with the deep sequencing data. It is interesting that there are more differentially expressed miRNAs in Wm/Sq than in Wm/BG. We found 12 miRNA families showed up- or down-regulation at least two-fold change only in watermelon grafted onto squash rootstock. For example, miR160a and miR2916 were in higher abundance in Wm/Sq than in Wm/Wm. Similarly, miR159a, miR172, miR395a and miR397 were identified to be less abundant in Wm/Sq than in Wm/Wm.

In addition, we also found that 15 (5 higher abundant and 10 lower abundant) and 30 (7 higher abundant and 23 lower abundant) novel miRNAs showed significant differences in Wm/BG and Wm/Sq compared with Wm/Wm, respectively (Fig. 5; Table S4). Out of these miRNAs, 8 novel miRNAs (cla-miR16, cla-miR54, cla-miR57, cla-miR59, cla-miR60, cla-miR65, cla-miR66, cla-miR80) were co-regulated in both Wm/BG and Wm/Sq. This suggests that novel miRNAs are involved in species-specific miRNA regulatory mechanisms in grafted-watermelon.

To gain insight into possible roles of the differently expressed miRNAs, their target genes were investigated. We found that the potential target genes included various transcriptional factors and other genes involved in a wide range of biological processes (Table 3). It implied that plant developmental processes might be affected after watermelon grafted onto different rootstocks. Base on the characteristic of their target genes, the differentially expressed miRNAs could be classified into four major categories. The first category includes 6 miRNA families- miR159, miR169, miR171, miR172, miR319 and miR396-which target transcription factors involved in regulating of gene expression and signal transduction. The second category includes miR160, miR167 and miR393, which are involved in direct response to stress or external stimuli. MiR160 and miR167 target auxin response factors ARF. Plant auxin regulates many important agronomical aspects of plant growth and development as well as adaptive to stress conditions. MiR393 target the messages of F-box proteins, which were also involved in auxin signaling and recently reported to be differentially regulated in response to cold, dehydration, salinity, and ABA treatments in *Arabidopsis* seedlings [17]. The third category includes miR395, miR397, miR398 and miR408, whose target genes are hydrolase and oxidoreductase-coding genes, like sulfate adenylyltransferase, laccases, Cu/Zn SOD and xanthine dehydrogenase. These proteins play role in adaptive responses to various stresses. The last category includes miR477, miR827, miR1661 and miR2916, which are less conserved and their functional regulation is not yet clear.

**Table 3:** Different expressed known miRNAs identified in watermelon grafted onto bottle gourd and squash rootstocks.

MiRNA	Family	*log2 (Wm/BG)/(Wm/Wm)	*log2(Wm/Sq)/(Wm/Wm)	Putative target
**miR159a**	miR159	−0.35	−1.04 ↓	MYB family transcription factor
**miR160a**	miR160	0.42	1.05 ↑	Auxin response factor
**miR167a**	miR167	−0.12	6.09 ↑	Auxin response factor
**miR167c**	miR167	0.23	−21.20 ↓	Auxin response factor
**miR169a**	miR169	0.23	1.12 ↑	NFY/MtHAP2-1 family transcription factor
**miR171a**	miR171	1.29 ↑	5.89 ↑	GRAS family transcription factor
**miR172**	miR172	0.40	−1.40 ↓	AP2 family transcription factor
**miR319a**	miR319	1.58 ↑	−17.82 ↓	TCP family transcription factor
**miR319c**	miR319	−0.06	−14.97 ↓	TCP family transcription factor
**miR393**	miR393	−0.26	−1.01 ↓	F-box protein
**miR395a**	miR395	−0.94	−5.30 ↓	Sulfate adenylyltransferase
**miR396**	miR396	0.35	7.33 ↑	GRF family transcription factor
**miR396a**	miR396	−3.54 ↓	−8.07 ↓	GRF family transcription factor
**miR397**	miR397	−0.90	−3.31 ↓	Laccases, Beta-6-tubulin
**miR398a**	miR398	5.45 ↑	1.95 ↑	CSD
**miR408**	miR408	−1.08 ↓	−4.08 ↓	Xanthine dehydrogenase
**miR477a**	miR477	8.26 ↑	9.85 ↑	GAI-like protein 1
**miR827**	miR827	−0.44	−1.61 ↓	P-loop containing nucleoside triphosphate hydrolases superfamily like protein
**miR166l**	miR166l	15.75 ↑	16.96 ↑	Class III homeodomain-leucine zipper
**miR2916**	miR2916	0.23	2.02 ↑	no target

* Log2 ratio of normalized miRNA expression in grafted watermelon compared with control; ↑and ↓:up- and down-regulated, respectively.

## Discussion

Gene regulation through sequence-specific interaction between miRNAs and their target genes offers an accurate mechanism for plant growth and response to environment stimuli. In parts of the world, horticulture is performed on unsuitable, marginal land, which causes various physiological and pathological disordered leading to severe crop losses. Grafting as an environment-friendly technique is widely used in commercial production to improve plant growth and tolerance to biotic and abiotic stresses. The physiological processes implicated in the graft-mediated regulation in various horticultural plants have received much attention, however, the molecular mechanism involved in this process remains relatively unknown. Therefore, extensive efforts are being made for unraveling genetic mechanisms, including discovering the role of miRNAs in this system. The identification of entire sets of miRNAs and their targets will lay a foundation for elucidation of the complex miRNA-mediated regulatory roles in graft system that control plant growth, development and other physiological processes. As an important food and agricultural commodities worldwide, watermelon is widely cultivated and most of them are grafted in China. The advent of high-throughput sequencing technologies has dramatically expanded the capacity of sRNA exploration, and in turn provided an efficient way to identify non-conserved, low accumulation, species-specific as well as conserved miRNAs on a large scale. The recent completion of the sequence of the watermelon genome provides a powerful resource for identification of watermelon miRNA.

In this study, we applied Solexa deep sequencing platform for in-depth characterization of the miRNA in grafted watermelons (watermelon was grafted on to bottle gourd and squash rootstocks, self-grafted watermelon was as control). Bottle gourd ‘Yongzhen’ (*Lagenaria siceraria*) and squash ‘Shintozwa’ (*Cucurbita maxima*×*Cucurbita moschata*) were used as rootstocks. Both of them are the most representative commercial ones used as rootstocks in China and they have different effects on plant growth and tolerance to environmental stress. Based on the sequence of the watermelon genome, 39 known miRNAs belonging to 30 miRNA families as well as 80 novel candidate miRNA were detected in grafted-watermelon for the first time. A wide range of characteristics was featured in these identified known miRNAs in watermelon. Similar to previous study, most of the identified known miRNAs families are highly evolutionarily conserved across multiple plant species (Table 2). To date, more than 21 families were found in at least 20 plant species, and they were conserved between dicots and monocots [40]. In long evolutionary timescales, well-conserved miRNA families have retained homologous target interactions and performed analogous molecular functions in plant kingdom [41]. It suggests that conservation consist their basically function for normal growth and development of plants. For example, most conserved miRNAs like miR156, miR159, miR160, miR164, miR167, miR169, miR171, miR172, miR319, miR396 and miR4414 target diverse transcription factors such as SBPs, MYBs, ARFs, NACs, NFY subunits, GRASs, AP2-like factors, TCPs, GRFs and WRKYs, and the miRNA-guided regulation of these transcription factor is critical for plant development and stress responses and may act as the main nodes in gene expression networks. In addition to well-conserved miRNAs, some less-conserved miRNAs like miR477, miR530, miR1310 and miR2916 were also detected by this study. It seems likely that these miRNAs relatively recently evolved, and play important roles in more species-specific way in plant growth and development [12]. The majority of these highly conserved miRNAs have relatively high expression abundance across Wm/BG, Wm/Sq and Wm/Wm. It is suggested that evolutionary conservation has a correlation with the expression level. The miRNAs were sequenced with a wide range from less than 10 times to thousands times and even tens of thousands times. This varied abundance of the miRNA families suggested that miRNAs with different biological functions would be differentially regulated.

More and more miRNAs have been identified by high-throughput sequencing from many plant species, proving that miRNAs are evolving rapidly. Each plant species has its specific miRNAs that are not present in others, even closely related species [42]. In this study, using minimal folding free energy index (MFEI) as criteria, we chose 80 out of more than seven hundred (data not shown) candidate miRNAs with MFEI >0.9 and reads count >50 to believed that those were most likely true novel miRNAs. These novel miRNA precursors had negative folding free energies (−218.5 to −17.5 kcal mol^−1^) with an average of about −59.2 kcal mol^−1^, and this was similar to the computational values of *Arabidopsis thaliana* miRNA precursors (−57 kcal mol^−1^) [43]. Based on BLASTn search against the watermelon genome, almost all of the novel miRNA are potentially generated from single loci and, as commonly observed in other plants, but not from transcriptional clusters. Dissimilar to conserved miRNAs, the novel miRNAs abundance in our database is at lower level, and they might be involved in more specific processes in watermelon. This is in agreement with previous study [29] that species-specific miRNAs are believed to be recently evolved and expressed at levels lower than those of strictly conserved miRNAs. Further functional analyses of these novel miRNAs will yield interesting and useful information on their role in signaling and development in grafted watermelon plants.

By comparing the expression level of miRNAs in different rootstocks grafted samples to control sample, a total of 17 out of 30 known miRNA families exhibited altered expression in watermelon after grafted on to bottle gourd or squash rootstocks. Notably, there were 5 miRNA families co-regulated in both graft systems. For example, miR396 and miR408 were highly reduced, while miR398 were highly induced in both Wm/BG and Wm/Sq, indicating that they might be involved in the pathway shared in the response to different rootstocks. Interestingly, there were more differentially expressed miRNAs in Wm/Sq than in Wm/BG. We found 12 miRNA families showed up- or down-regulated at least two-fold change only in watermelon grafted onto squash rootstock. For example, miR160a and miR2916 were in higher abundance in Wm/Sq than in Wm/Wm. Similarly, miR159a, miR172, miR395a and miR397 were identified to be less abundant in Wm/Sq than in Wm/Wm. The results of this work suggest that some miRNAs seem to be specific to squash rootstock mediated grafting. These finding strongly implicates that grafting could indeed affect the expression level of miRNAs in scion. Moreover, watermelon grafted onto bottle gourd rootstock or squash rootstock exhibits differently. The differentially expressed miRNAs in grafted watermelon may be related with the regulatory role of grafting in plant development and adaptive responses to stresses. The predicted target genes for the graft-associated miRNAs encode proteins of diverse function, most of them being transcription factors, and others were associated with signal transduction, metabolism, growth and development processes (Table 3). Metabolites, hormones, proteins and nucleic acids may act as systemic signals that provide an efficient exchange of information between tissues. For most of the conserved miRNAs, it is expected their targets are also conserved. For example, our result showed that miR396 was down-regulated in both of Wm/BG and Wm/Sq compared with Wm/Wm. MiR396 has been known to target GRF transcription factors and be involved in the regulation of growth and cell proliferation in leaves [44]. However how grafting change miR396 expression level to regulate the cell cycle needs more study. Recently, miR396 was also found to be responsive to high salinity, drought, and cold stress in *Arabidopsis* [44], and miR396 families was observed down-regulated in salt-shocked maize roots [45], thus supporting the hypothesis of a role for miR396 in grafted watermelons in the adaptive response to abiotic stress. Another miRNA, miR398, was down-regulated in both Wm/BG and Wm/Sq. Previous studies showed that miR398 was transcriptionally down-regulated to release it targets suppression of CSD1 and CSD2 genes in response to oxidative stress [24]. Further insight into the role of miR398 has been observed that miR398 plays an important dual but opposite role during normal growth conditions and abiotic stress. The miR398-guided regulation might be crucial for the expression of optimal CSD1 and CSD2 that in turn regulate the levels of superoxide or other ROS required for signaling in a spatial- and temporal-specific manner under normal growth conditions [46]. So whether miR398 in grafted watermelon would be down regulated and improve stress tolerance under stress conditions need further research. Moreover, among miRNAs that had rootstock-specific regulation, miR160a and miR167a were up-regulate in Wm/Sq, but not in Wm/BG. Their putative targets are members of ARFs, which are critical elements in regulation of physiological and morphological mechanisms mediated by auxin that may contribute to environmental stress adaptation [37]. Similarly, miR159 and miR172 were down-regulate in Wm/Sq. Previous studies showed that transgenic *Arabidopsis* overexpressing gibberellin-regulated miR159 showed a delay in flowering time [47]. By contrast, overexpression of miR172, which targets AP2-type transcription factors, resulted in early flowering [48]. Additionally, miR172 regulates Flowering Locus T (FT) protein in *Arabidopsis* [49], the systemic effect of this miRNA might be mediated by FT. Moreover, two miR156 target, *SPL9* and *SPL10*, regulate the levels of miR172 and the effect of *SPL9* on vegetative development was reported [50], suggesting that either SPL proteins or downstream factors, including miR172 or FT, might be involved in the regulating flowering time in graft watermelon. As we known, miR159 and miR172 are also involved in response to abiotic stresses. Interestingly, previous studies showed that many of the miRNAs target genes involved in growth and development are stress-regulated as well.

It is noteworthy that the effects of graft on plant growth and development and even adaptation to stress should not only due to the simple physiological change, the potential signaling roles of miRNAs by the long-distance regulation of gene expression might be involved in this system. The vascular system, especially the phloem, provides the pathway for the systemic translocation of macromolecules [30]. The certainty that RNA transcripts and small RNA are present, mobile in the phloem, and likely function as information molecules is one of the most exciting new prospects in plant biology. Long-range signaling has a major role in the regulation and coordination of different biological processes, such as flowering, leaf and vascular development, resource allocation, pathogen defence, environmental stress response and silencing of RNAs [51], [52]. The phloem stream of higher plants contains a multitude of small molecules and macromolecules, including proteins, mRNAs and small RNAs [53]. The first discovery that miRNAs might be systemic molecules in plants was the finding of endogenous miRNAs in phloem exudates of pumpkin, cucumber, castor bean and yucca [54], and later in oilseed rape [55] and white lupin [56]. Previous studies have revealed more than one hundred miRNAs, belonging to tens of families, in exudates of several plants, such as miRNAs involved in development (e.g. miR156, miR159, miR173) or responses to nutrient stresses (miR395, miR398 and miR399) [57]. However, it is the fact that very few of phloem exudate miRNAs seem to move, it is expected that miRNA trafficking be tightly regulated. Previous study indicated that miR399 from the over-expressing scion moved across the graft junction to the wild-type rootstock to cleave *PHO2* mRNA directly in roots [58]. Similar to miR399, miR395 was able to cross graft junctions in response to S starvation, using WT/*hen1-1* grafts [59]. Only these two miRNAs have been elucidated clearly that they can move long distance to response to nutrient availability. Additionally, the presence of miR172 in vascular bundles and the graft transmissibility of its effect on potato tuberization were observed by Martin et al. [60], they suggest that either miR172 might be mobile or it regulates long-distance signal to induce tuberization. By comparing the miRNA repertoires among Wm/Wm, Wm/BG and Wm/Sq, we found that some miRNAs were differently expressed in the combinations, so we hypothesize that some miRNAs might be involved in the long-distance signal pathway in this graft system. Rootstocks and scions can communicate in both directions; however, root-to-shoot or shoot-to-root signaling requires further studies. Thus, our results may serve as a point for future studies investigating the long-distance regulation of miRNAs in grafted watermelon. Testing whether miRNAs can also move between species could open a new area of research.

### Conclusion

Grafting is an effective agricultural approach to improve plant growth and resistance to stresses. Based on the high throughput Solexa sequencing, we discovered 30 known miRNA families and 80 novel miRNAs in watermelon. Compared with self-grafted watermelon, 20 (5 known miRNA families and 15 novel miRNAs) and 47 (17 known miRNA families and 30 novel miRNAs) miRNAs are expressed with significant difference with higher or lower abundance in watermelon grafted onto bottle gourd and squash, respectively. The identification of entire sets of miRNAs and their different expression patterns in watermelon grafted onto different rootstocks will lay a foundation for elucidation of the complex miRNA mediated regulatory system which involved in agronomical important grafted watermelon and may play visually important role in plant growth, development, and response to stresses.

## Materials and Methods

### Sample Preparation and Total RNA Extraction

Watermelon (*Citrullus lanatus* L. cv. IVSM9) was grafted onto two rootstocks: Bottle gourd ‘Yongzhen’ (*Lagenaria siceraria*) (abbreviated as Wm/BG), and squash ‘Shintozwa’ (*Cucurbita maxima*×*Cucurbita moschata*) (Wm/Sq), and watermelon plants grafted onto watermelon (Wm/Wm) were used as control. Plants were cultivated in the growth chamber at a photosynthetic photon flux density (PPFD) of 600 µmol m^−2^ s^−1^ with a photoperiod of 12 h, 25°C (day) and 17°C (night) temperature, and between 50% and 85% humidity. At the two true-leaf stage, the part above cotyledons of the scion (Fig. 1) was collected and stored at −80°C until further use. For Solexa sequencing, 3 plants for each combination were pooled together, and total RNA was extracted from pooled samples of Wm/Wm, Wm/BG and Wm/Sq separately with TRIzol reagent (Invitrogen, Carlsbad, CA, USA) according to the manufacturer’s instructions. RQ1 RNase-Free DNase (Promega, Madison, WI, USA) was used to remove genomic DNA contamination. The quality and integrity of RNAs were examined using an Agilent 2100 Bioanalyzer (RNA Nano Chip, Agilent).

### Small RNA Library Construction and Solexa Sequencing

Small RNA fragments (14–30 bases) were isolated from the total RNA pool with a Novex 15% TBE-Urea gel (Invitrogen). The purified small RNAs were then ligated with 5′ adaptors (Illumina, San Diego, CA, USA). To remove these unligated adaptors, the ligation products (40–60 bases in length) were gel purified on a Novex 15% TBE-Urea gel. Subsequently, a 3′ adaptor (Illumina) was ligated to the 5′ ligation products. After gel purification on a Novex 10% TBE-Urea gel (Invitrogen), RNA fragments with adaptors at both ends (70–90 bases in length) were reversely transcribed, and the resulting cDNAs were subjected to 15 PCR cycles using the adaptor primers. The amplification products (around 90 bases) were excised from a 6% TBE-Urea gel (Invitrogen). The purified cDNAs library was used directly for cluster generation and sequencing analysis by an Illumina/Solexa G1 sequencer.

### Identification of Known and Novel miRNAs

Sequencing reads were extracted from the image files generated by Illumina Genome Analyzer and then processed to produce digital-quality data. The subsequent procedures performed with the Illumina data were summarizing data production, evaluating sequencing quality, calculating length distribution of small RNA reads and masking low quality reads and adaptor sequences. The remaining reads were analysed by BLAST against cucurbit species mRNAs, Rfam (ftp.sanger.ac.uk/pub/databases/Rfam) and Repbase (http://www.girinst.org/) to discard mRNA, rRNA, tRNA, snRNA, snoRNA and repeat sequences. By aligning to the Rfam database, we could annotate a variety of annotations to each sRNA sequence, such as rRNA, tRNA, snRNA and snoRNA. By aligning to the Repbase database, we could annotate repeat sequences. Subsequently, 15–26 nt non-annotated sequences were analyzed by BLAST against miRBase 17.0 (http://mirbase.org/). Solexa sequences with identical or related (one mismatch) sequences from mature miRNAs were identified as known miRNAs [61].

Then, the remaining sequences were searched by BLAST against watermelon genome (provided by National Engineering Research Center for Vegetables, Beijing Academy of Agriculture and Forestry Sciences, China) to identify novel miRNAs. The mappable sequences were then folded into a secondary structure using an RNA-folding program mFold3.6. The criteria used to annotate potential miRNAs was as decribed by previously [61]. Sequences that met the following described criteria were then considered to be miRNA precursors. The strict criteria included: 1) the sequence could fold into an appropriate stem-loop hairpin secondary structure, 2) the small RNA sits in one arm of the hairpin structure, 3) less than 12 nucleotides in one bulge in stem and more than 18 base pairs in the stem region of the predicted hairpin, 4) no more than 8 nucleotides and 4 biased errors in one bulge and no more than 2 biased bulges and 6 errors in mature region of the predicted hairpin, 5) more than 15 base pairs in mature region and 80 percent of mature region in stem, and 6) predicted secondary structure has higher minimal folding free energy index (MFEI = [(MFE/length of the RNA sequence)×100]/(G+C)%) and negative minimal folding free energy.

### Target Gene Prediction

Previous studies have proved that all miRNAs fulfilling the function of post-transcriptional gene regulation by binding to the target mRNA sequences in one or more perfect or near-perfect complementary site (s), bring convenience to predict plant miRNA targets simply using gene-homology search. We used the newly identified miRNA to search their putative targets against annotated watermelon cds database (ICuGI) with the TagetFinder 1.6.

### Expression Pattern of miRNAs among Wm/Wm, Wm/BG and Wm/Sq

To investigate the differentially expressed miRNAs among Wm/Wm, Wm/BG and Wm/Sq, firstly, each identified miRNAs read count was normalized to the total number of miRNA reads in each given sample and multiplied by a million. Normalized sequence counts were used for differential expression analysis with IDEG6 (http://telethon.bio.unipd.it/bioinfo/IDEG6_form/). The selection methods of differential expression were Audic and Claverie, Fisher’s exact test and chi-squared 2×2, with the selection threshold of 0.001 (http://telethon.bio.unipd.it/bioinfo/IDEG6_form/detail.html#AC). Then, if the change in normalized sequence counts was more than two folds, a specific miRNA was considered to be expressed significantly different.

### Verification of miRNAs by Stem-loop Quantitative Real-time PCR (RT-qPCR)

To validate the presence and expression of the identified miRNAs, 7 known miRNAs and 5 novel miRNAs with different expression patterns were selected for stem-loop RT-qPCR as described previously [62]. The total RNA was reverse-transcribed using miRNA specific stem-loop primers (Table S5). The reverse transcript reaction was performed with M-MLV (Takara, China) following the manufacturer’s protocol. Reverse transcript products were used as template for RT-qPCR with each gene-specific primers (Table S5) and all reaction were assayed in triplicates. The reactions were performed in ABI PRISM 7900HT (Applied Biosystems, USA) using Platinum SYBR Green qPCR SuperMix-UDG (Invitrogen, USA). PCR cycling began with template denaturation and hot start Taq activation at 95°C for 2 min, then 40 cycles of 95°C for 15 sec, and 60°C for 30 sec performed and data collected during each cycle at the 60°C extension step. The U6 snRNA was selected as a reference gene for normalization. The relative expression level of miRNA was calculated according to the method of Livak and Schmittgen [63].

## Supporting Information

Table S1 **Novel miRNAs identified in this study.**
(XLSX)

Table S2 **Predicted targets for known miRNAs.**
(XLSX)

Table S3 **Predicted targets for novel miRNAs.**
(XLSX)

Table S4 **Different expressed miRNAs in Wm/BG and Wm/Sq compared with Wm/Wm.**
(XLSX)

Table S5 **Primer sequences used for RT-qPCR.**
(XLSX)
